# Severity of Betel Quid Use Disorders (BUD) Among Adult Indian Betel Quid Chewers

**DOI:** 10.1111/odi.70118

**Published:** 2025-10-10

**Authors:** Stefano Petti, Roopa Shamsundar Rao, Pallavi Deshmukh, Sowmya Gujjar Vishnu Rao, Ashok Lingappa, Krishna Sireesha Sundaragiri, Chandni Shekhawat, Tejavathi Nagaraj, Surendra Lakshminarayana, Saman Warnakulasuriya

**Affiliations:** ^1^ Department of Public Health and Infectious Diseases Sapienza University Rome Italy; ^2^ Faculty of Dental Sciences Ramaiah University of Applied Sciences Bangalore Karnataka India; ^3^ H.K.E S's S. Nijalingappa Institute of Dental Sciences & Research Kalaburgi Karnataka India; ^4^ Institute of Dental Sciences Bareilly International University Bareilly Uttar Pradesh India; ^5^ Bapuji Dental College and Hospital Davangere Karnataka India; ^6^ RUHS College of Dental Sciences Jaipur Rajasthan India; ^7^ Sri Rajiv Gandhi College of Dental Sciences Bangalore Karnataka India; ^8^ King's College London London UK

**Keywords:** betel quid use disorders, common factor analysis, India, prevalence

## Abstract

**Objective:**

Oral cancer is a major public health problem in India, with the highest number of cases and deaths in the world, and Betel Quid (BQ) chewing is responsible for almost 50% of cases. BQ Use Disorder (BUD) prevalence and severity have never been investigated among Indian BQ chewers.

**Methods:**

In total, 1281 adult routine BQ chewers attending five dental healthcare centers were interviewed using a pretested 11‐item BUD questionnaire based on the DSM‐5 substance use disorder (SUD) diagnostic criteria. Demographic, lifestyle, and other BQ‐related variables also were collected.

**Results:**

Females were almost 80%, and two‐thirds were rural residents. BQ was used prevalently with friends; the average initiation age was 24 years, and two‐thirds used BQ daily. Severe and mild‐to‐moderate BUD was detected in 49% and 45% of individuals, respectively. The average BUD score (range 0–11) was 5.2. Sex‐standardized severe BUD prevalence was 54.9% (95% confidence interval, 49.8%–59.7%). Female sex, older age, being married, using BQ with friends, and adding tobacco were inversely associated with severe BUD. Multicollinearity was noted among these factors.

**Conclusion:**

Severe BUD was highly prevalent in Indian BQ chewers. Intervention programs aimed at assisting chewers to quit the habit should account for this dramatic situation. Appropriate measures should be taken to effectively influence cessation rates.

## 1 Introduction

The international Agency for Research on Cancer (IARC) in 2004 classified betel quid (BQ) and its primary ingredient Areca nut as Class 1 carcinogens (IARC Working Group on the Evaluation of Carcinogenic Risks to Humans 2004). BQ and areca nut use are recognized as major risk factors for oral cancer in most countries in South and Southeast Asia and particularly in India (Guha et al. 2014; Ray and Gupta 2025). BQ is the fourth most popular psychoactive agent used worldwide. The Asian BQ Consortium evaluated the intercountry prevalence and practices of its use in South, Southeast, and Eastern Asia regions (Lee et al. 2011). The same group also reported that the criteria for substance use dependence provided by the American Psychiatric Association in their Diagnostic and Statistical Manual of Mental Disorders, Fourth Edition (DSM‐4) (American Psychiatric Association 1994) apply to a significant proportion of BQ users in Asian communities (Lee et al. 2014). Matching with the DSM‐5 (American Psychiatric Association 2013) substance use disorder (SUD) diagnostic criteria, the authors developed a DSM‐5 BQ Use Disorder (BUD) questionnaire consisting of 11 questions for use among Asian BQ users (Lee et al. 2018). Using this questionnaire, the Asian BQ Consortium conducted a joint study to evaluate the effects of BUD on oral health among six nations in South Asia, but India was not included in this study. Since there are no data on BUD for any Indian population, and only sparse data exist on BQ use dependence (Prabhu and Acharya 2025; Kumar et al. 2021; Sadath et al. 2022), the country with the highest number of oral cancer cases and deaths, further research is warranted.

The primary aim of this study was to investigate the prevalence proportion of individuals with severe BUD among adult regular BQ chewers in India. The secondary aim was to investigate the variables potentially associated with severe BUD.

## 2 Materials and Methods

A multicenter cross‐sectional study was designed.

### 2.1 Settings

Subjects attending five outpatient dental clinics (Table S1, Figure S1) were eligible. This hospital‐based sampling method introduced a certain degree of selection bias, because these subjects were not representative of the general population of BQ chewers (Delgado‐Rodríguez and Llorca 2004). Thus, in order to limit this bias, only patients who did not seek for care for oral diseases associated with heavy BQ use, specifically, diseases of the oral mucosa, were considered (Lee, Ko, et al. 2012). The survey was made between 2024 and 2025.

### 2.2 Eligible Individuals

Eligible individuals were adult patients aged 18+ years, referring to dental clinics, excluding those referring to oral medicine and pathology units. Patients were preliminarily inquired about BQ usage to exclude non‐users and former users; regular BQ users were considered self‐identified current BQ chewers who reported chewing for more than 1 year and at a current rate of at least three times per week (Lee, Chang, et al. 2012) (Lee et al. 2018).

Eligible individuals were asked to sign the informed consent.

### 2.3 Sampling Selection Procedure

Hospital‐based, non‐probability (convenience), cluster sample was chosen. Although this procedure limited the generalizability of the reported results due to selection bias, this shortcoming was partly controlled by trying to get the highest possible participation rate (Tyrer and Heyman 2016).

The assumptions to calculate the sample size were: 50% severe BUD prevalence in BQ users, pre‐estimated using previous studies (Lee, Chang, et al. 2012; Lee, Ko, et al. 2012; Lee et al. 2018), 10% margin of error (i.e., expected prevalence between 40% and 60%), 95% confidence limit, and finite population size of 1,000,000 individuals. The following formulas were applied:

$$X=\left[\left(Z_{\alpha /2}\right)\times p\times \left(1-p\right)\right]/{\mathrm{MOE}}^{2}$$

where *X* is the sample size estimated without correction for finite population size; *Z*_α/2_ is the critical value of the Normal distribution at *α*/2 (with the 95% confidence level, *α* is 0.05 and the critical value was 1.96); MOE is the margin of error; and *p* is the assumed sample proportion;

$$n=N\times X/\left[X\times \left(N-1\right)\right]$$

where *n* is the required sample size using the single random sampling method, and *N* is the finite population size. With the above assumptions *n* = 96.03.

Cluster sampling introduced the clustering effect, as individuals in the same cluster tended to be more alike in terms of certain characteristics. This effect reduced the precision of the prevalence proportion estimate compared to the single random sampling method. This changed precision was measured using the design effect (DEFF), which assesses the homogeneity within a cluster and the variability between clusters (World Health Organization et al. 2020). The required sample size for single random sampling must be multiplied by the DEFF.

The DEFF was calculated with the formula:

$$\text{DEFF}=1+\left[\left(\dot{n}-1\right)\times \mathrm{ICC}\right]$$

where *ṅ* is the average number of units per cluster, set at 200 individuals, and ICC is the intra‐class correlation, a measure of the similarity of clusters in a given characteristic. The ICC value for socio‐demographic and lifestyle characteristics in the Indian context was estimated as the average of the ICC's extracted from published data (Oommen et al. 2019; Dwivedi et al. 2023). With average ICC = 0.055, DEFF = 11.945, and the required sample size was *n* = 1147 individuals.

This value was divided by 0.90 assuming a participation rate of 90%. Therefore, the resulting sample size was 1245, with at least 200 individuals included in each cluster.

### 2.4 Questionnaire

Participating subjects were interviewed by trained clinicians. The first part of the questionnaire included demographic and lifestyle characteristics. The second part of the questionnaire was finalized at performing BUD diagnosis. BUD is not formally identified by the eleventh Revision of the International Classification of Diseases (ICD‐11) in the Section of “Disorders due to substance use or addictive behaviors” (World Health Organization 2024), and there are no universally acknowledged diagnostic criteria. According to the DSM‐5 (American Psychiatric Association 2013), Substance Abuse and Substance Dependence are combined into SUD, and a questionnaire‐based screening tool has been designed for this condition (Hasin et al. 2013). The BUD 11‐item questionnaire, developed and validated by the Asian BQ Consortium study and based on these criteria, produced a score ranging between 0 and 11. Individuals were classified as having no BUD for scores 0–1, mild BUD for scores 2–3, moderate BUD for scores 4–5, and severe BUD for scores > 5 (Lee et al. 2018).

### 2.5 Severe BUD Prevalence Assessment

The first issue was to assess whether the present sample was representative of the underlying study population of BQ users. For this reason, a large cross‐sectional study among adult Indian patients investigating BQ use prevalence was considered as reference (Gupta and Jain 2024); this sample was stratified according to demographic variables that were dichotomized, assuming that each category of the dichotomized variable included roughly one half of the general population. More specifically, subjects were split according to gender (males and females), residence (urban and rural residents), and education (secondary school diploma or higher and primary school diploma or lower). Then, within each variable, the Prevalence Ratio between the two categories was calculated using the category with the highest prevalence as the numerator. The proportions of BQ using individuals in the two categories resulted from the formulas:

- Proportion of BQ users in the category with higher prevalence = (Prevalence Ratio)/[(Prevalence Ratio) + 1]
- Proportion of BQ users in the category with lower prevalence = 1/[(Prevalence Ratio) + 1]

For example, if the underlying proportion of BQ using males in India was double that of females, the Prevalence Ratio was 2, and it was expected that a representative sample of BQ users included 2/3 males and 1/3 females.

This procedure allowed building standard reference populations of adult Indian BQ users, according to the three demographic variables: gender, residence, and education. Following the previous example, a standard reference population of 1000 adult Indian BQ users according to gender included 667 males (i.e., 2/3) and 333 females (i.e., 1/3).

In order to investigate whether the present sample was representative of the study population of adult Indian BQ users, the proportions of patients according to the three dichotomized demographic variables were assessed, and 95% confidence intervals (95% CI's) were assessed using the Wilson score method (Newcombe 1998). This confidence interval was compared to the reference proportion of the standard adult population of BQ users, and if the latter value did not fall within the 95% CI, with a further 5% tolerance, then the present sample was considered not representative of the study population of adult Indian BQ users for that variable.

In the event that the sample was not representative, overall severe BUD prevalence could not be assessed cumulatively, and a standardization was made according to the three demographic variables, using the newly generated standard reference populations of adult Indian BQ users. Three standardizations were made according to gender, residence, and education.

This standardization process allowed for the assessment of reliable BUD prevalence estimates even in the absence of representative samples of BQ users.

### 2.6 Factors Associated With Severe BUD

The outcome variable was severe BUD and was dichotomized into severe vs. no/mild/moderate. The BUD questionnaire score also was considered as a secondary outcome.

The set of explanatory variables included demographic, lifestyle variables, and variables related to BQ use. The assumption of Normality for these variables, necessary for the application of further statistical tests, was investigated by assessing skewness and kurtosis. Normality was rejected for skewness values outside ±2 and kurtosis values outside ±7. Non‐normally distributed variables were either transformed into dichotomous, normalized, or excluded from the analysis (West et al. 1995).

### 2.7 Common Factor Analysis

Many of the explanatory variables were likely inter‐correlated, a problem that could negatively impact the multivariate analysis. Therefore, the correlation between variables was investigated. The Pearson's correlation coefficient “r” was assessed, and if the number of r's with P‐value < 0.05 resulted higher than one third, the level of inter‐correlation between variables was considered too high. In this case, Common Factor Analysis (CFA) was used to obtain a smaller set of new variables (called “Factors”), with a lower degree of inter‐correlation that incorporated the original variables.

The eigenvalues of the generated Factors were assessed. Eigenvalue measures how much of the common variance of the original variables was explained by the Factor. Factors with eigenvalue > 1 were considered. With such a high number of explanatory variables, the newly generated Factors were also likely inter‐correlated. Thus, orthogonal rotation with the Varimax method was used to decrease the level of correlation between Factors. The meaningfulness of the generated Factors was investigated with the correlation coefficients, called Factor Loadings, between the original variables and each Factor. Variables with Factor Loading ≥ 0.4 were considered highly correlated with the Factor; variables with Factor Loading ranging between ≥ 0.15 and < 0.4 were moderately correlated (Pippi et al. 2025; Petti and Scully 2010; Passariello et al. 2017).

The generated Factors were an expression of the explanatory variables that were moderately/highly correlated with the Factor. Thus, the CFA individuated a set of BQ using Profiles (i.e., the Factors) that were characterized by certain properties (i.e., the explanatory variables with moderate/high Factor Loadings) and were not inter‐correlated.

### 2.8 Association With Severe BUD

A preliminary investigation of the association between the explanatory variables and severe BUD was made with logistic regression analysis. The unadjusted odds ratios (ORs) with 95% CIs were assessed. Then, multiple regression analysis was also made using all the explanatory variables. The set of explanatory variables was tested for collinearity and multicollinearity through the Pearson's correlation matrix (r) and the Variance Inflation Factor (VIF). Collinearity was considered high for *r* > 0.5, moderate for *r* > 0.1; multicollinearity was considered high for VIF > 5, moderate for VIF > 1 (VIF = 1 is the ideal situation with regression *R* ^2^ = 0).

Due to likely substantial collinearity/multicollinearity, which would produce spurious and inflated OR estimates, the less biased approach proposed in the present study was to investigate the association between the BQ using Profiles that were designed to minimize inter‐correlation and severe BUD. Thus, instead of identifying specific single risk factors for severe BUD, which were, however, potentially unreliable, this methodology allowed for identifying the characteristics of individuals at higher risk of severe BUD.

Using the same methodology used to investigate the dietary patterns (Flood et al. 2008), the Factor scores of each BQ using Profile, generated by the CFA for each subject, were divided into quartiles, with Quartile 1 (Q1) including individuals that adhered to that Profile most.

For each Profile, the OR for severe BUD was assessed. Logistic regression analysis was used with Q4 as reference, and Q3, Q2, Q1 as incremental levels of adherence to the Profile. The extended Mantel–Haenszel *χ* ^2^ test for linear trend was applied to test whether the OR estimates for each quartile were proportional and there was an increasing/decreasing trend in the association between that Profile and severe BUD. As all explanatory variables were included in the generated Profiles, and since the CFA rotation procedure produced non‐intercorrelated Factors, the multiple regression analysis was not necessary. Anyway, the Pearson's correlation matrix (*r*) and the Variance Inflation Factor (VIF) were assessed; if collinearity and multicollinearity were close to zero, the multiple regression analysis was not performed.

Statistical analyses were performed using MedCalc (Version 20.104; MedCalc Software Ltd), StatView (Version 5.0.1; SAS Institute Inc), VassarStats (Website for Statistical Computation, available at http://vassarstats.net/), and OpenEpi (Version 3.01, available at https://www.openepi.com/Menu/OE_Menu.htm).

## 3 Results

### 3.1 General Characteristics of the Sample and the Five Clusters

Overall participation rate was 70% (Table S1). The main reasons for non‐participation were the denial to disclose personal information, the lack of incentives, a typical problem in the Indian context, and incompleteness of data at the early stages of the anamnesis. Patients were 98% Indians, almost 80% were females, two thirds were rural residents, and 75% were married, while almost one half were non‐skilled manual workers (Table 1, Table S2).

**TABLE 1:** Demographic characteristics of the whole sample and of patients with and without severe BUD.

	Whole sample	Severe BUD	Non‐severe/no BUD
Dental Clinic*			
Govt. Dental Institute	18.0% (230)	43.5% (100)	56.5% (130)
Private Dental Institute DVG	18.0% (230)	7.0% (16)	93.0% (214)
Private Dental Institute Bareilly	18.0% (230)	72.2% (166)	27.8% (64)
Private Dental Institute RG, BNG	28.2% (361)	52.6% (190)	47.4% (171)
Private Dental Institute GUL	18.0% (230)	67.8% (156)	32.2% (74)
Ethnicity			
Indian	98.1% (1257)	48.7% (612)	51.3% (645)
Malaysian, Nepalese	1.9% (24)	66.7% (16)	33.3% (8)
Age*			
18–25 years	16.9% (217)	68.2% (148)	31.8% (69)
26–35 years	39.6% (379)	47.8% (181)	52.2% (198)
36–45 years	24.4% (312)	44.6% (139)	55.4% (173)
46–60 years	24.5% (314)	43.0% (135)	57.0% (179)
> 60 years	4.6% (59)	42.4% (25)	57.6% (34)
Gender**			
Male	21.1% (270)	59.3% (160)	40.7% (110)
Female	78.9% (1011)	46.3% (468)	53.7% (543)
Residence			
Rural	63.4% (812)	47.4% (385)	52.6% (427)
Urban	36.6% (469)	51.8% (243)	48.2% (226)
Education*			
Illiterate	16.9% (217)	58.5% (127)	41.5% (90)
Literate	14.8% (190)	67.9% (129)	32.1% (61)
Primary school	38.0% (487)	33.7% (164)	66.3% (323)
Secondary school	20.4% (261)	55.2% (144)	44.8% (117)
University	9.8% (126)	50.8% (64)	49.2% (62)
Occupation**			
Professional	3.7% (47)	74.5% (35)	25.5% (12)
Non‐manual worker	6.1% (78)	65.4% (151)	34.6% (27)
Skilled manual worker	20.1% (257)	47.1% (121)	52.9% (136)
Non‐skilled manual worker	46.7% (598)	47.0% (281)	53.0% (317)
Unemployed	10.4% (133)	49.6% (66)	50.4% (67)
Student	7.4% (95)	42.1% (40)	57.9% (55)
Retired	3.0% (38)	42.1% (16)	57.9% (22)
Homemaker	2.7% (35)	51.4% (18)	48.6% (17)
Marital status*			
Married	74.9% (960)	44.5% (427)	55.5% (533)
Single	20.7% (265)	62.6% (166)	37.4% (99)
Separated	1.6% (20)	60.0% (12)	40.0% (8)
Widowed	2.0% (25)	72.0% (18)	28.0% (7)
Divorced	0.9% (11)	45.5% (5)	55.5% (6)

*Note:* Difference between BUD categories assessed with *χ* ^2^ test.

* *p*‐value < 0.0001.

** *p*‐value < 0.001.

BQ with tobacco (26.6%), gutka (24.0%), nut alone (21.8%), and pan masala (18.4%) were the products that were more frequently used (Table 2, Table S3). 91% of the sample used only one product, 36% declared that they drink alcoholic beverages, 30% that they use SLT alone, and 23% smoked.

**TABLE 2:** Characteristics of BQ use in the whole sample and of patients with and without severe BUD.

	Whole sample	Severe BUD	Non‐severe/no BUD
Nut alone			
No	78.2% (1002)	79.9% (502)	76.6% (500)
Yes	21.8% (279)	20.1% (126)	23.4% (153)
BQ without tobacco*			
No	85.9% (1100)	81.8% (514)	89.7% (586)
Yes	14.1% (181)	18.2% (114)	10.3% (67)
BQ with tobacco*			
No	73.4% (940)	83.8% (526)	63.4% (414)
Yes	26.6% (341)	16.2% (102)	36.6% (239)
Pan masala**			
No	81.6% (1045)	78.0% (490)	85.0% (555)
Yes	18.4% (236)	22.0% (138)	15.0% (98)
Gutka*			
No	76.0% (974)	70.7% (444)	81.2% (530)
Yes	24.0% (307)	29.3% (184)	18.8% (123)
Khara**			
No	99.3% (1272)	70.7% (444)	40.7% (110)
Yes	0.7% (9)	29.3% (184)	53.7% (543)
Others***			
No	95.5% (1223)	94.1% (591)	96.8% (632)
Yes	4.5% (58)	5.9% (37)	3.2% (21)
Total products			
1	90.7% (1162)	88.9% (558)	92.5% (604)
2	8.4% (108)	9.9% (62)	7.0% (46)
3	0.9% (11)	1.3% (8)	0.5% (3)
Smokeless tobacco (SLT)*			
No	70.3% (901)	62.9% (395)	77.5% (506)
Yes	29.7% (380)	37.1% (233)	22.5% (147)
Smokeless tobacco (SLT) with slaked lime			
No	86.5% (1108)	86.8% (545)	86.2% (563)
Yes	13.5% (173)	13.2% (83)	13.8% (90)
Smoking (cigarettes, beedi)**			
No	76.8% (984)	81.4% (511)	72.4% (473)
Yes	23.2% (297)	18.6% (117)	27.6% (180)
Alcoholic drinks			
No	64.3% (824)	64.0% (402)	64.6% (422)
Yes	35.7% (457)	36.0% (226)	35.4% (231)

*Note:* Difference between BUD categories assessed with *χ* ^2^ test.

* *p*‐value < 0.0001.

** *p*‐value < 0.01.

*** *p*‐value < 0.05.

Two thirds of the sample used BQ daily (Table 3, Table S3), between one and four intakes per occasion. BQ was used prevalently with friends; the average initiation age was 24 years.

**TABLE 3:** BQ use in the whole sample and in patients with and without severe BUD.

	Whole sample	Severe BUD	Non‐severe/no BUD
BQ use frequency			
Weekly	37.6% (482)	60.4% (379)	64.3% (420)
Daily	62.4% (799)	39.7% (249)	35.7% (233)
BQ amount in each intake occasion*			
1–4	48.4% (620)	55.7% (350)	41.3% (270)
5–9	20.1% (257)	19.4% (122)	20.7% (135)
10–15	13.2% (169)	13.1% (82)	13.3% (87)
> 15	18.3% (235)	11.8% (74)	24.7% (161)
BQ use**			
Alone	5.0% (64)	5.7% (36)	4.3% (28)
With family	13.6% (174)	17.4% (108)	10.0% (65)
With friends	77.8% (996)	74.2% (466)	81.2% (530)
Mixed	3.7% (47)	2.7% (17)	4.6% (30)
Swallow BQ juice*			
No	51.8% (663)	46.0% (289)	57.3% (374)
Yes	48.2% (618)	54.0% (339)	42.7% (279)
BQ starting age*			
Mean age (years)	23.7 ± 7.1	21.8 ± 6.8	24.8 ± 7.1
Use duration			
Mean duration (years)	8.4 ± 5.9	7.7 ± 5.4	9.0 ± 6.2

*Note:* Difference between BUD categories assessed with *χ* ^2^ test (proportions) and Student's *t*‐test for unpaired samples (means).

* *p*‐value < 0.0001.

** *p*‐value < 0.001.

### 3.2 Severe BUD Prevalence

According to the BUD questionnaire, 49% of patients were classified as having severe BUD (Table 4), while another 45% as mild to moderate BUD. The average score was 5.2 points. Prevalence in Cluster 2 was 7% while in Cluster 3 was 72% (Table S4), with the average score ranging between 3.1 (Cluster 2) and 6.0–6.2 (Clusters 3 and 5).

**TABLE 4:** BQ use disorders (BUD) in the whole sample and in patients with and without severe BUD.

	Whole sample	Severe BUD	Non‐severe/no BUD
BUD categories			
No	6.2% (80)		
Mild	22.2% (285)		
Moderate	22.5% (288)		
Severe	49.0% (628)		
BUD score (mean)			
Total score*	5.2 ± 2.5	7.3 ± 1.4	3.2 ± 1.4
Reinforcement construct*	2.5 ± 1.1	3.2 ± 0.9	1.9 ± 1.0
Social impairment*	1.1 ± 1.0	1.8 ± 1.0	0.5 ± 0.7
Stimulation construct*	0.8 ± 0.8	1.3 ± 0.7	0.4 ± 0.6
Pharmacologic symptoms*	0.8 ± 0.7	1.1 ± 0.6	0.4 ± 0.6

*Note:* Difference between BUD categories assessed with Student's *t*‐test for unpaired samples (means).

* *p*‐value < 0.0001.

The BQ use Prevalence Ratios extracted from the reference study were 1.92 for gender (males more prevalent than females), 1.92 for residence (rural more prevalent than urban), and 2.26 for education (primary school or lower, more prevalent). Therefore, the standard reference population of 1000 adult Indian BQ users included 658 males and 342 females according to gender; 658 rural and 342 urban residents according to residence; and 693 patients with primary school diploma or lower and 307 patients with secondary school diploma or higher according to education (Table S5). The proportions of rural/urban residents and patients with lower/higher education in the present sample were similar to the proportions of the standardized reference population (Table S6); thus, no adjustment according to these demographic variables was necessary. Conversely, the departure from the standard population according to gender was very important. Therefore, standardization for gender was necessary.

As expected, the standardizations for residence and education provided similar severe BUD prevalence estimates as the crude prevalence estimate, with wider confidence intervals (Table 5). Conversely, the standardization according to gender resulted in a higher prevalence estimate that was 54.9%, although the 95% CIs of the non‐standardized and standardized estimates were overlapping, suggesting that the difference between the two estimates was not statistically significant. Nevertheless, since the gender distribution in the present sample was largely different from the usual distribution observed in BQ users, the gender‐standardized estimate was considered the most reliable.

**TABLE 5:** Prevalence of severe BUD in the overall sample and standardized prevalence proportion according to gender, residence, and education.

Standardization	Proportion (*n*/*N*)	Prevalence (95% CI)	Expected cases in the standard population (95% CI)
Non‐standardized	628/1281	49.0% (46.3%–51.8%)	N/A
Gender			
Males	160/270	59.3% (53.3%–65.0%)	390.2 (350.7–427.7)
Females	468/1011	46.3% (43.2%–49.4%)	158.3 (147.7–168.9)
Standardized		54.9% (49.8%–59.7%)	
Residence			
Rural	385/812	47.4% (44.0%–50.9%)	311.9 (289.5–334.9)
Urban	243/469	51.8% (47.3%–56.3%)	177.2 (161.8–192.5)
Standardized		48.9% (45.1%–52.7%)	
Education			
Lower	420/894	47.0% (43.7%–50.3%)	325.7 (302.8–348.6)
Higher	208/387	53.8% (48.9%–58.7%)	165.2 (150.1–180.2)
Standardized		49.1% (45.3%–52.9%)	

### 3.3 Risk Factors for Severe BUD

Non‐normally distributed demographic and lifestyle variables were normalized, dichotomized, or not considered at all, as reported in Table S7. At the end of this stage, there were 23 remaining variables. The multivariate analysis showed that lower education, non‐manual working compared to manual/non‐working, non‐married marital status, BQ use alone/within family compared to use with friends, starting BQ use at a younger age, use duration lower than 10 years, swallowing BQ juice, and the use of SLT without tobacco and alcohol were significantly associated with a higher probability of severe BUD (Table S8). However, the level of collinearity between these variables was very high, as almost 60% of the Pearson's correlation coefficients between the 23 variables were statistically significant with *p*‐value < 0.05 (Table S9), and an average moderate multicollinearity was also found (Table S10). Therefore, the degree of inter‐correlation between the explanatory variables was considerably high, and such an event inflated the regression coefficients and/or produced potentially spurious associations. This result was expected because, in the present sample, there were large groups of individuals with similar characteristics. For example, 26% of the sample were females older than 35 years, with a primary school degree or no degree at all, and manual working. Assuming that BUD prevalence was significantly higher or lower in this stratum compared to the other strata of the sample, it would be impossible to speculate an independent role of female gender, higher age, low education, and poor occupation in BUD development. This high inter‐correlation made the CFA necessary.

The CFA generated 10 Factors with eigenvalue higher than 1.0 that explained two thirds of the overall common variance of the original variables (Table S9). From the analysis of the Factor Loadings, 10 Profiles were delineated with specific characteristics (Table 6). The principal Profile that accounted for more than 12% of the common variance was characterized by aged married women who started using BQ at an advanced age and used it for more than 10 years, did not use gutka and smokeless tobacco, and were manual working or not working. Individuals in the Q1 were those who adhered to the Profile characteristics most, while those in Q4 were those who adhered least.

**TABLE 6:** Individual profiles generated by the common factor analysis.

Factor (Profile)	Characteristics
Profile 1	*Primary*: Female, aged and married *Secondary*: manual/not working, started using BQ at advanced age, use BQ from > 10 years and do not use gutka, and do not use SLT
Profile 2	*Primary*: rural with low education, prefer gutka, use BQ daily, swallow BQ juice *Secondary*: use BQ with friends, do not use nut alone
Profile 3	*Primary*: do not use BQ with friends, use other BQ products *Secondary*: urban, with low education, do not use nut alone, consume few BQ intakes, use SLT with slaked lime
Profile 4	*Primary*: do not use nut alone, use BQ with tobacco *Secondary*: high education, started using BQ at older age, consume many BQ intakes daily
Profile 5	*Primary*: use nut alone, do not use BQ without tobacco *Secondary*: rural, use BQ with tobacco, started using BQ at older age, consume many BQ intakes
Profile 6	*Primary*: consume many BQ intakes, use SLT with slaked lime *Secondary*: started using BQ at older age, swallow BQ juice, do not use SLT alone
Profile 7	*Primary*: do not use SLT alone, smoker *Secondary*: started using BQ at older age, do not drink alcohol
Profile 8	*Primary*: rural, manual worker/not working, use BQ from > 10 years *Secondary*: use BQ weekly
Profile 9	*Primary*: do not use SLT alone, drink alcohol *Secondary*: urban, do not swallow BQ juice
Profile 10	*Primary*: do not use pan masala *Secondary*: use BQ with friends, use nut alone, use BQ with tobacco, use BQ from < 10 years, consume many BQ intakes, do not use SLT with slaked lime

*Note:* Primary characteristics are the demographic/lifestyle variables with Factor Loading ≥ 0.4, denoting high correlation with the Profile. Secondary characteristics are variables with Factor Loading ranging between ≥ 0.15 and < 0.4, denoting moderate correlation with the Profile.

The Profile that showed the strongest association with BUD was Profile 1, inversely associated, with ORs that were progressively declining from Q3 to Q1, and Q1 individuals that had one third the probability of Q4 individuals to show severe BUD. Profiles 5, 6, 7, and 8 also were inversely associated with severe BUD with decreasing OR trends, while Profile 3 was the only profile that was directly associated with severe BUD and with an increasing OR trend from Q3 to Q1 (Table 7). Profile 3 was characterized by urban individuals with low education, who used non‐conventional BQ products and did not use nut alone, consumed few BQ intakes in each using occasion, stayed alone or within family, and also used SLT with slaked lime (Table 6).

**TABLE 7:** Logistic regression analysis of the association between BQ using Profiles and severe BUD; OR trend analysis with Mantel–Haenszel (MH) *χ* ^2^ test for linear trend.

BQ using Profile	Quartile	OR	95% CI	Severe BUD cases (%)	Total	MH *χ* ^2^ (*p*)
Profile 1	Q4	1.00	N/A	193 (60.3%)	320	42.16 (< 0.0001)
	Q3	0.77	0.57–1.06	174 (54.0%)	322	
	Q2	0.55	0.40–0.75	148 (45.4%)	326	
	Q1	0.37	0.27–0.51	113 (36.1%)	313	
Profile 2	Q4	1.00	N/A	167 (52.0%)	321	1.29 (0.25)
	Q3	0.73	0.54–1.00	140 (44.3%)	316	
	Q2	0.67	0.49–0.92	136 (42.1%)	323	
	Q1	1.25	0.92–1.71	185 (57.6%)	321	
Profile 3	Q4	1.00	N/A	121 (38.1%)	318	16.61 (< 0.0001)
	Q3	1.68	1.23–2.30	164 (50.8%)	323	
	Q2	1.80	1.31–2.45	164 (52.6%)	312	
	Q1	1.96	1.43–2.68	179 (54.6%)	328	
Profile 4	Q4	1.00	N/A	166 (50.9%)	326	31.58 (< 0.0001)
	Q3	1.53	1.12–2.09	195 (61.3%)	318	
	Q2	1.13	0.83–1.54	171 (53.9%)	317	
	Q1	0.41	0.30–0.57	96 (30.0%)	320	
Profile 5	Q4	1.00	N/A	185 (57.1%)	324	23.61 (< 0.0001)
	Q3	0.87	0.64–1.19	171 (53.6%)	319	
	Q2	0.63	0.46–0.86	149 (45.6%)	327	
	Q1	0.49	0.36–0.68	123 (39.6%)	311	
Profile 6	Q4	1.00	N/A	180 (55.2%)	326	17.24 (< 0.0001)
	Q3	0.93	0.68–1.27	169 (53.5%)	316	
	Q2	0.73	0.53–0.99	151 (47.2%)	320	
	Q1	0.54	0.40–0.74	128 (40.1%)	319	
Profile 7	Q4	1.00	N/A	180 (55.9%)	322	17.15 (< 0.0001)
	Q3	0.90	0.66–1.23	170 (53.3%)	319	
	Q2	0.66	0.48–0.90	147 (45.5%)	323	
	Q1	0.56	0.41–0.76	131 (41.3%)	317	
Profile 8	Q4	1.00	N/A	187 (57.5%)	325	16.26 (< 0.0001)
	Q3	0.72	0.53–0.99	159 (49.5%)	321	
	Q2	0.66	0.48–0.90	149 (47.2%)	316	
	Q1	0.53	0.39–0.72	133 (41.7%)	319	
Profile 9	Q4	1.00	N/A	202 (63.1%)	320	6.99 (0.008)
	Q3	0.37	0.27–0.51	125 (38.9%)	321	
	Q2	0.45	0.33–0.61	140 (43.3%)	323	
	Q1	0.60	0.44–0.83	161 (50.8%)	317	
Profile 10	Q4	1.00	N/A	175 (54.0%)	324	8.58 (0.003)
	Q3	0.85	0.62–1.15	158 (49.8%)	317	
	Q2	0.87	0.64–1.18	161 (50.5%)	319	
	Q1	061	0.45–0.83	134 (41.7%)	321	

*Note:* For each Profile, individuals in the lowest quartile (Q4) were the reference group, while individuals in the other quartiles were the exposure groups with gradually higher adherence to the Profile.

## 4 Discussion

This survey found that the estimated prevalence proportion of severe BUD adjusted for sex was 55% (95% CI 50%–60%), higher than the crude prevalence estimate (49%, 95% CI 46%–52%), although this difference was not statistically significant (Table 5). In addition, the profiles with the lowest probability of severe BUD were Profile 1 made up of aged and married females and Profile 3 made up of individuals that use BQ with tobacco and with friends (Tables 6 and 7).

### 4.1 Study Limits and Generalizability

Hospital‐based sampling method and participation rate may have hampered the generalizability of the severe BUD prevalence estimate. In general, hospital‐based cross‐sectional studies, like the present survey, are not representative of the general population, due to differences in lifestyle and health literacy between individuals who attend and do not attend dental healthcare facilities, as in the example of preventive services (Wong et al. 2021; Netuveli et al. 2006). Assuming a similar situation in the present study, prevalence of unhealthy lifestyle in non‐attending individuals would be higher than in the sampled patients, due to limited resources and awareness toward oral health problems (Petti 2010), with consequent underestimation of severe BUD. Similarly, health‐related surveys show important differences between participating and non‐participating individuals, with males, the elderly, the least educated, and immigrants showing lower likelihood to agree to participate and/or complete the questionnaires, particularly in rural and socioeconomically deprived areas (Lyngsøe et al. 2024). The present problems likely generated a certain degree of participation bias, suggesting that the reported data are underestimates of the true severe BUD prevalence.

In the Indian context, however, the situation could be different, as at least nine out of 10 individuals have minimal health literacy (Mathias et al. 2023), and even among attending graduates and post‐graduates, the level of health literacy is poor (D'Cruz and Shankar Aradhya 2013; Ramandeep et al. 2014). This suggests that the differences between attending and non‐attending, participating and non‐participating individuals must be lower than expected, with limited impact of selection bias.

The multi‐center nature of this survey could capture different realities (Figure S1). Namely, three dental healthcare facilities (Table S1) were located in Karnataka, an affluent South‐Western State with just 5% of people in poverty, while the remaining two were in Uttar Pradesh, a Northern State, and Rajasthan, another Northern State, with 17% and 14% of people below the poverty line, respectively. In addition, public and private dental healthcare settings located in urban and rural areas were deliberately chosen, thus diversifying the characteristics of the sampled individuals and increasing the result generalizability (Sprague et al. 2009). A second countermeasure applied in the present study was that the methodology of rate standardization was used to estimate the prevalence proportion. Actually, for non‐representative samples in analytical epidemiology (i.e., in the study of associations between variables), the use of weights is recommended (Haneuse et al. 2009), but no similar measures are reported in descriptive epidemiology to adjust the results when sample representativeness is unclear. For this reason, an acknowledged methodology was used, mediated from the assessment of adjusted population incidence and mortality rates.

In conclusion, although selection bias could not be excluded, the peculiar Indian context and the methodological countermeasures probably limited the impact of this bias, which, if present, would have led to underestimate the true severe BUD prevalence.

### 4.2 Comparison With Other Studies

A recent systematic review reported the proportion of BUD among BQ chewers in South and Southeast Asia ranged from 55.2% to 99.3% (Ko et al. 2025). Paradoxically, in India, the country with the highest number of oral cancer cases and deaths (International Agency for Research on Cancer (IARC) 2022) that are largely due to exposure to BQ chewing (Senevirathna et al. 2023), the information regarding BQ use disorders, addiction, and dependence is sparse, and before 2020, there were no data at all (Lee et al. 2011; Lee, Chang, et al. 2012; Lee, Ko, et al. 2012).

A literature search was made using PubMed and the keywords (“betel quid” AND “India” AND “dependence”/”addiction”/”use disorders”) and only three single‐center surveys were found, none investigated BUD directly. One from Karnataka, made on 400 adult BQ users recruited in healthcare settings (participation rate not reported), found that 65% of participants were dependent on BQ and reported that dependence was more prevalent among elderly males with low literacy levels (Prabhu and Acharya 2025). Another survey was made in Assam, a poor State with per‐capita income 40% lower than in the whole of India and 32% of people below the poverty line. The survey included 479 BQ‐using adults recruited in healthcare settings (participation rate 96%) and reported that although 64% were aware that BQ use causes oral cancer, only 18% ever tried to quit chewing and 15% thought of quitting (Kumar et al. 2021). Finally, another survey among indigenous tribes was conducted in Kerala, the most affluent Indian State with just 1% of people below the poverty line and included 2186 individuals (participation rate not reported) and found BQ use prevalence proportion of 48% with 16% of subjects consuming BQ more than five times daily, a proxy of BUD (Sadath et al. 2022).

The present multi‐center survey suggests that BQ use disorders are present in almost all BQ users (93.8%, Table S4) with minimal differences across Indian States, and severe BUD is suggested in at least one half of the users. These data are partly corroborated by these single‐center surveys.

### 4.3 Clinical Relevance

This survey among Indian BQ users examined the prevalence and severity of BUD based on DSM‐5 symptoms. The study demonstrates that close to 50% of the BQ users surveyed had a severe BUD. The IARC handbook on Oral Cancer Prevention (IARC Working Group on the Evaluation of Cancer‐Preventive Interventions 2023) highlighted the crucial importance of promoting cessation programs among BQ users for advancing preventive strategies on oral cancer. Cessation of lifestyle risk factors such as smoking, drinking, and BQ using is a difficult task (S. Petti 2009; Lindson et al. 2024). Therefore, the impact and success of such programs will depend on the prevalence of BUD among the studied population. Those found with severe BUD are unlikely to benefit from attempts to self‐quit the BQ habit due to their strong addiction to the BQ habit. Any intervention planned needs to take account of the severity of BUD in the population to achieve a successful quit rate. Establishing a BUD‐based risk assessment is recommended to identify high‐risk groups that may need special attention and appropriate behavioral and pharmaco‐therapies when organizing oral cancer prevention programs in India. For those determined to have severe BUD scores (> 6 out of 11 items), it may be necessary to engage specialist services when appropriate.

## Author Contributions

**Stefano Petti:** conceptualization, writing – original draft, methodology, validation, writing – review and editing, software, formal analysis, project administration, data curation, supervision. **Roopa Shamsundar Rao:** data curation, investigation, writing – review and editing. **Pallavi Deshmukh:** investigation. **Sowmya Gujjar Vishnu Rao:** investigation. **Ashok Lingappa:** investigation. **Krishna Sireesha Sundaragiri:** investigation. **Chandni Shekhawat:** investigation. **Tejavathi Nagaraj:** investigation. **Surendra Lakshminarayana:** investigation. **Saman Warnakulasuriya:** conceptualization, writing – original draft, writing – review and editing, methodology, project administration, data curation, supervision, validation.

## Ethics Statement

The research protocol was approved by the Institutional Ethics Committee of the Faculty Dental Surgery, Ramaiah University of Applied Sciences, Bangalore (India), and the Institutional Review Boards of the other participating institutions (documents are available upon request to the authors).

## Conflicts of Interest

The authors declare no conflicts of interest.

## Supporting information


**Data S1:** odi70118‐sup‐0001‐Supinfo.pdf.

## Data Availability

Data are available upon reasonable request to the authors.

## References

[odi70118-bib-0001] American Psychiatric Association . 1994. Diagnostic and Statistical Manual of Mental Disorders. 4th ed. American Psychiatric Association.

[odi70118-bib-0002] American Psychiatric Association . 2013. Diagnostic and Statistical Manual of Mental Disorders, Fifth Edition. American Psychiatric Association.

[odi70118-bib-0003] D'Cruz, A. M. , and M. R. Shankar Aradhya . 2013. “Health Literacy Among Indian Adults Seeking Dental Care.” Dental Research Journal 10, no. 1: 20–24. 10.4103/1735-3327.111760. 23878559 PMC3714819

[odi70118-bib-0004] Delgado‐Rodríguez, M. , and J. Llorca . 2004. “Bias.” Journal of Epidemiology and Community Health 58, no. 8: 635–641. 10.1136/jech.2003.008466. 15252064 PMC1732856

[odi70118-bib-0005] Dwivedi, L. K. , B. Mahaptra , A. Bansal , J. Gupta , A. Singh , and T. K. Roy . 2023. “Intra‐Cluster Correlations in Socio‐Demographic Variables and Their Implications: An Analysis Based on Large‐Scale Surveys in India.” SSM Population Health 21: 101317. 10.1016/j.ssmph.2022.101317. 36589273 PMC9798159

[odi70118-bib-0006] Flood, A. , T. Rastogi , E. Wirfält , et al. 2008. “Dietary Patterns as Identified by Factor Analysis and Colorectal Cancer Among Middle‐Aged Americans.” American Journal of Clinical Nutrition 88: 176–184. 10.1093/ajcn/88.1.176. 18614739 PMC2495056

[odi70118-bib-0007] Guha, N. , S. Warnakulasuriya , J. Vlaanderen , and K. Straif . 2014. “Betel Quid Chewing and the Risk of Oral and Oropharyngeal Cancers: A Meta‐Analysis With Implications for Cancer Control.” International Journal of Cancer 135, no. 6: 1433–1443. 10.1002/ijc.28643. 24302487

[odi70118-bib-0008] Gupta, J. , and V. Jain . 2024. “Knowledge, Attitude, and Practices of Areca Nut and Betel Quid Chewing Among the Adult Population – A Questionnaire‐Based Cross‐Sectional Study.” SRM Journal of Research in Dental Sciences 15: 15–21. 10.4103/srmjrds.srmjrds_127_23.

[odi70118-bib-0009] Haneuse, S. , J. Schildcrout , P. Crane , J. Sonnen , J. Breitner , and E. Larson . 2009. “Adjustment for Selection Bias in Observational Studies With Application to the Analysis of Autopsy Data.” Neuroepidemiology 32, no. 3: 229–239. 10.1159/000197389. 19176974 PMC2698450

[odi70118-bib-0010] Hasin, D. S. , C. P. O'Brien , M. Auriacombe , et al. 2013. “DSM‐5 Criteria for Substance Use Disorders: Recommendations and Rationale.” American Journal of Psychiatry 170, no. 8: 834–851. 10.1176/appi.ajp.2013.12060782. 23903334 PMC3767415

[odi70118-bib-0011] IARC Working Group on the Evaluation of Cancer‐Preventive Interventions . 2023. Oral Cancer Prevention. IARC.

[odi70118-bib-0012] IARC Working Group on the Evaluation of Carcinogenic Risks to Humans . 2004. “Betel‐Quid and Areca‐Nut Chewing and Some Areca‐Nut Derived Nitrosamines.” IARC Monographs on the Evaluation of Carcinogenic Risks to Humans 85: 1–334. 15635762 PMC4781453

[odi70118-bib-0013] International Agency for Research on Cancer (IARC) . 2022. “Cancer Today. Data Visualization Tools for Exploring the Global Cancer Burden in 2022.” https://gco.iarc.fr/today/en.

[odi70118-bib-0014] Ko, A. M. , P. W. Wu , W. T. Lin , and C. H. Lee . 2025. “Betel‐Quid Addictive Use Disorders and Oral Potentially Malignant Disorders and Oral Cancer in South, Southeast, and East Asia: A Systematic Review and Meta‐Analysis.” Oral Diseases 31, no. 5: 1517–1530. 39164987 10.1111/odi.15106

[odi70118-bib-0015] Kumar, A. , K. Oswal , R. Singh , et al. 2021. “Assessment of Areca Nut Use, Practice and Dependency Among People in Guwahati, Assam: A Cross‐Sectional Study.” E Cancer Medical Science 15: 1198. 10.3332/ecancer.2021.1198. PMC8043683 33889207

[odi70118-bib-0016] Lee, C. H. , S. L. Chiang , A. M. Ko , et al. 2014. “Betel‐Quid Dependence Domains and Syndrome Associated With Betel‐Quid Ingredients Among Chewers: Asian Multi‐Country Evidence.” Addiction 109, no. 7: 1194–1204. 10.1111/add.12530. 24650227

[odi70118-bib-0017] Lee, C. H. , A. M. Ko , S. Warnakulasuriya , et al. 2011. “Intercountry Prevalences and Practices of Betel‐Quid Use in South, Southeast and Eastern Asia Regions and Associated Oral Preneoplastic Disorders: An International Collaborative Study by Asian Betel‐Quid Consortium of South and East Asia.” International Journal of Cancer 129, no. 7: 1741–1751. 10.1002/ijc.25809. 21128235

[odi70118-bib-0018] Lee, C. H. , A. M. Ko , F. M. Yang , et al. 2018. “Association of DSM‐5 Betel‐Quid Use Disorder With Oral Potentially Malignant Disorder in 6 Betel‐Quid Endemic Asian Populations.” JAMA Psychiatry 75, no. 3: 261–269. 10.1001/jamapsychiatry.2017.4307. 29417149 PMC5885949

[odi70118-bib-0019] Lee, C. H. , A. M. Ko , C. F. Yen , et al. 2012. “Betel‐Quid Dependence and Oral Potentially Malignant Disorders in Six Asian Countries.” British Journal of Psychiatry 201, no. 5: 383–391. 10.1192/bjp.bp.111.107961. 22995631

[odi70118-bib-0020] Lee, C. Y. , C. S. Chang , T. Y. Shieh , and Y. Y. Chang . 2012. “Development and Validation of a Self‐Rating Scale for Betel Quid Chewers Based on a Male‐Prisoner Population in Taiwan: The Betel Quid Dependence Scale.” Drug and Alcohol Dependence 121, no. 1–2: 18–22. 10.1016/j.drugalcdep.2011.07.027. 21955360

[odi70118-bib-0021] Lindson, N. , A. R. Butler , H. McRobbie , et al. 2024. “Electronic Cigarettes for Smoking Cessation: A Cochrane Review.” Dental Cadmos 92, no. 6: 466–479. 10.19256/d.cadmos.06.2024.05.

[odi70118-bib-0022] Lyngsøe, S. , S. Lophaven , R. Jepsen , T. Holmager , A. Janssens , and E. Lynge . 2024. “Non‐Participation in a Health Examination Survey in a Rural‐Provincial Area of Denmark—Results From the Lolland‐Falster Health Study (LOFUS).” Scandinavian Journal of Public Health 52, no. 8: 951–959. 10.1177/14034948231206879. 37953717 PMC11626842

[odi70118-bib-0023] Mathias, E. G. , V. S. Dhyani , J. B. Krishnan , U. Rani , N. Gudi , and S. Pattanshetty . 2023. “Community Based Health Literacy Interventions in India: A Scoping Review.” Clinical Epidemiology and Global Health 22: 101310. 10.1016/j.cegh.2023.101310.

[odi70118-bib-0024] Netuveli, G. , A. Sheiham , and R. G. Watt . 2006. “Does the ‘inverse Screening Law’ Apply to Oral Cancer Screening and Regular Dental Check‐Ups?” Journal of Medical Screening 13, no. 1: 47–50. 10.1258/096914106776179836. 16569306

[odi70118-bib-0025] Newcombe, R. G. 1998. “Two‐Sided Confidence Intervals for the Single Proportion: Comparison of Seven Methods.” Statistics in Medicine 17, no. 8: 857–872. 10.1002/(sici)1097-0258(19980430)17:8<857::aid-sim777>3.0.co;2-e. 9595616

[odi70118-bib-0026] Oommen, A. M. , G. K. Mini , and K. George . 2019. “Intracluster Correlation Estimates From a World Health Organisation STEPwise Approach to Surveillance (STEPS) Survey for Cardiovascular Risk Factors in Vellore, Tamil Nadu, India.” Public Health 168: 102–106. 10.1016/j.puhe.2018.12.019. 30738282

[odi70118-bib-0027] Passariello, C. , P. Gigola , L. Testarelli , M. Puttini , S. Schippa , and S. Petti . 2017. “Evaluation of Microbiota Associated With Herpesviruses in Active Sites of Generalized Aggressive Periodontitis.” Annali di Stomatologia 8, no. 2: 59–70. 10.11138/ads/2017.8.2.071. 29299190 PMC5749375

[odi70118-bib-0028] Petti, S. 2009. “Lifestyle Risk Factors for Oral Cancer.” Oral Oncology 45, no. 4–5: 340–350. 10.1016/j.oraloncology.2008.05.018. 18674956

[odi70118-bib-0029] Petti, S. 2010. “Why Guidelines for Early Childhood Caries Prevention Could Be Ineffective Amongst Children at High Risk.” Journal of Dentistry 38, no. 12: 946–955. 10.1016/j.jdent.2010.09.002. 20837088

[odi70118-bib-0030] Petti, S. , and C. Scully . 2010. “Determinants of Oral Cancer at the National Level: Just a Question of Smoking and Alcohol Drinking Prevalence?” Odontology 98, no. 2: 144–152. 10.1007/s10266-010-0133-4. 20652793

[odi70118-bib-0031] Pippi, R. , M. L. Lauteri , and S. Petti . 2025. “Is Pre‐Operative Full Mouth Plaque Score Related to Inflammatory Complications in Lower Third Molar Surgery? Cohort Study.” Clinical Oral Investigations 29, no. 7: 364. 10.1007/s00784-025-06442-x. 40590983

[odi70118-bib-0032] Prabhu, A. , and S. Acharya . 2025. “Determinants of Betel Quid Dependence and Predictive Efficacy of Cessation Scales Among Rural Users in Southern India.” Journal of Ethnicity in Substance Abuse: 1–15. 10.1080/15332640.2025.2544282. 40779642

[odi70118-bib-0033] Ramandeep, G. , S. Arshdeep , K. Vinod , and P. Parampreet . 2014. “Oral Health Literacy Among Clients Visiting a Rural Dental College in North India–A Cross‐Sectional Study.” Ethiopian Journal of Health Sciences 24, no. 3: 261–268. 10.4314/ejhs.v24i3.10. 25183933 PMC4141230

[odi70118-bib-0034] Ray, C. S. , and P. C. Gupta . 2025. “Oral Cancer in India.” Oral Diseases 31, no. 5: 1439–1454. 10.1111/odi.14974. 38950053

[odi70118-bib-0035] Sadath, A. , K. Jose , K. M. Jiji , V. T. Mercy , G. Ragesh , and E. Arensman . 2022. “Prevalence and Determinants of Substance Use Among Indigenous Tribes in South India: Findings From a Tribal Household Survey.” Journal of Racial and Ethnic Health Disparities 9, no. 1: 356–366. 10.1007/s40615-021-00964-2. 33495925

[odi70118-bib-0036] Senevirathna, K. , R. Pradeep , Y. A. Jayasinghe , et al. 2023. “Effects of Areca Nut and Its Metabolites: A Review of the Experimental Evidence.” Clinics and Practice 13, no. 2: 326–346. 10.3390/clinpract13020030. 36961055 PMC10037666

[odi70118-bib-0037] Sprague, S. , J. M. Matta , M. Bhandari , et al. 2009. “Multicenter Collaboration in Observational Research: Improving Generalizability and Efficiency.” Journal of Bone and Joint Surgery. American Volume 91, no. Suppl 3: 80–86. 10.2106/JBJS.H.01623. 19411504

[odi70118-bib-0038] Tyrer, S. , and B. Heyman . 2016. “Sampling in Epidemiological Research: Issues, Hazards and Pitfalls.” BJPsych Bulletin 40, no. 2: 57–60. 10.1192/pb.bp.114.050203. 27087985 PMC4817645

[odi70118-bib-0040] West, S. G. , J. F. Finch , and P. J. Curran . 1995. “Structural Equation Models With Nonnormal Variables: Problems and Remedies.” In Structural Equation Modelling: Concepts, Issues, and Applications, edited by R. H. Hoyle , 56–75. Sage Publications; Thousand Oaks.

[odi70118-bib-0041] Wong, T. J. , Q. Li , V. Dodd , W. Wang , J. Bian , and Y. Guo . 2021. “Oral Cancer Knowledge and Screening Behaviour Among Smokers and Non‐Smokers in Rural Communities.” BMC Cancer 21, no. 1: 430. 10.1186/s12885-021-08198-5. 33879128 PMC8056680

[odi70118-bib-0042] World Health Organization . 2024. “ICD‐11. International Classification of Diseases 11th Revision.” https://icd.who.int/en.

[odi70118-bib-0043] World Health Organization , US Centers for Disease Control and Prevention , Nutrition International , and UNICEF . 2020. “Micronutrient Survey Manual.” https://mnsurvey.nutritionintl.org/.

