# Supplementary material for: Severity of Betel Quid Use Disorders (BUD) Among Adult Indian Betel Quid Chewers
Source: Oral Dis. 2025 Oct 10;32(6):1544–55. doi: 10.1111/odi.70118 (PMC13457683; doi:10.1111/odi.70118)
Supplement: Supplementary file 1 [file ODI-32-1544-s001.pdf]

Severity of Betel Quid Use Disorders (BUD) among adult Indian Betel Quid chewers

Supplementary Material

Figure S1. Prevalence of BQ chewing individuals among adult patients attending the five dental healthcare settings (clusters) during the survey (2024-2025).

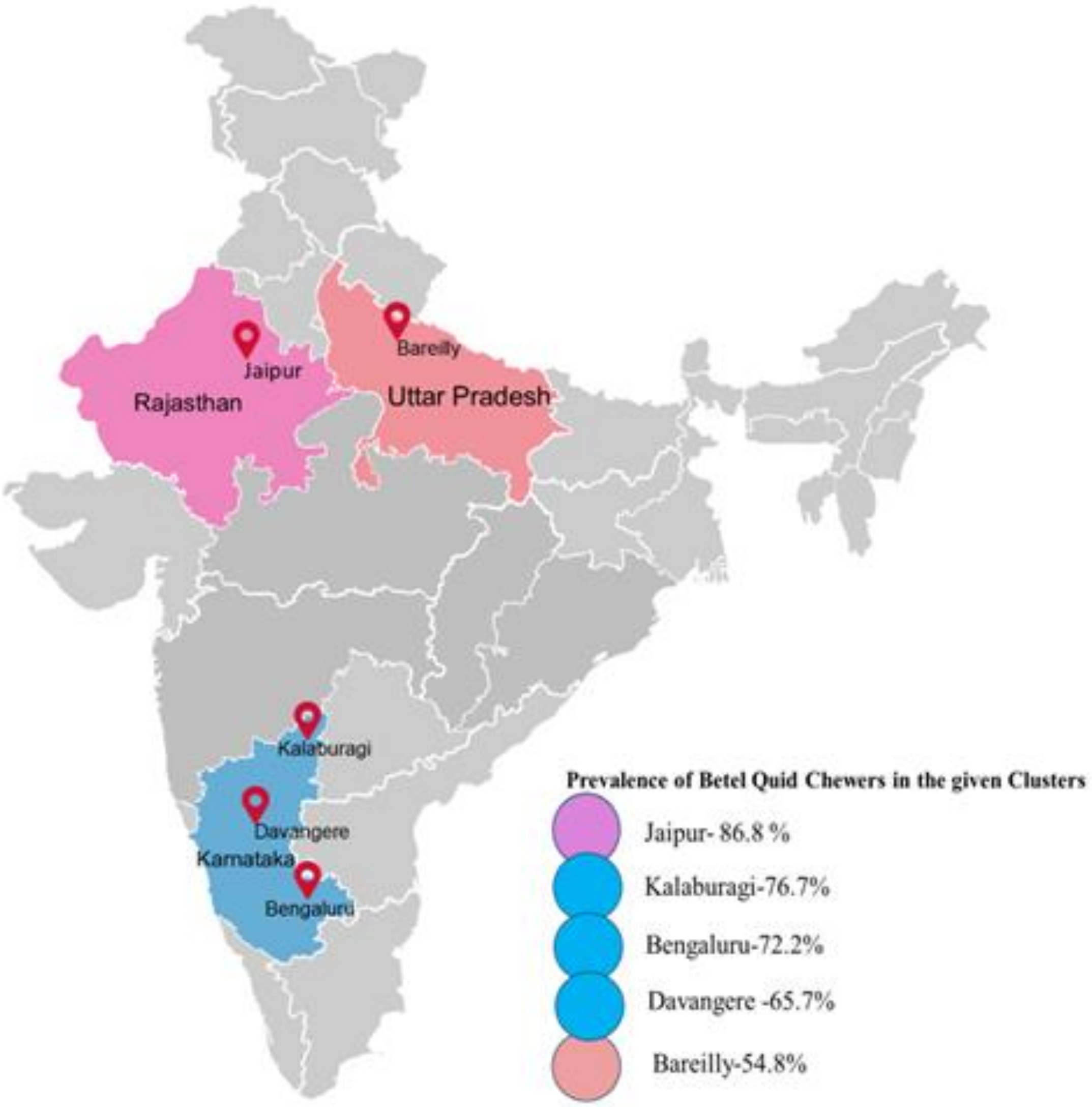

Table S1. Oral healthcare settings included in the present study and participation rates.

Cluster	Oral healthcare setting	Region	Participation rate
Cluster 1	Nijalingappa Institute of Dental Sciences and Research	Kalaburgi, Karnataka	76.7% (230/300)
Cluster 2	Bapuji Dental College and Hospital	Davangere, Karnataka	65.7% (230/350)
Cluster 3	Institute of Dental Sciences	Bareilly, Uttar Pradesh	54.8% (230/420)
Cluster 4	Rajiv Gandhi College of Dental Sciences and Hospital	Bangalore, Karnataka	72.2% (361/500)
Cluster 5	RUHS College of Dental Sciences	Jaipur, Rajasthan	86.8% (230/265)
Total			69.8% (1281/1835)

Table S2. Demographic characteristics of patients in the five clusters (i.e., the five Dental Clinics where patients were recruited), and statistical analysis of the differences between clusters assessed with the χ^2 test. Clusters that deviated from the overall frequency distributions contributing to more than one third the overall χ^2 value were reported.

Ethnicity

	Indian	Malaysian	Nepalese
Cluster 1	94.8% (218)	3.0% (7)	2.2% (5)
Cluster 2	100.0% (230)	0.0% (0)	0.0% (0)
Cluster 3	100.0% (230)	0.0% (0)	0.0% (0)
Cluster 4	96.7% (349)	1.1% (4)	2.2% (8)
Cluster 5	100.0% (230)	0.0% (0)	0.0% (0)
Total	98.1% (1257)	0.9% (11)	1.0% (13)

Difference between groups χ^2_{8df}=34.7; P-value<0.0001.

Highest contribution to χ^2 value from Cluster 1 (15.8).

Age groups

	18-25 years	26-35 years	36-45 years	46-60 years	>60 years
Cluster 1	21.7% (50)	30.0% (69)	30.4% (70)	14.8% (34)	3.0% (7)
Cluster 2	3.0% (7)	43.9% (101)	20.4% (47)	20.9% (48)	11.7% (27)
Cluster 3	13.0% (30)	26.5% (61)	26.1% (60)	29.6% (68)	4.8% (11)
Cluster 4	8.3% (30)	21.1% (76)	29.9% (108)	36.8% (133)	3.9% (14)
Cluster 5	43.5% (100)	31.3% (72)	11.7% (27)	13.5% (31)	0.0% (0)
Total	16.9% (217)	39.6% (379)	24.4% (312)	24.5% (314)	4.6% (59)

Difference between groups χ^2_{16df}=277.6; P-value<0.0001.

Highest contribution to χ^2 value from Cluster 5 (132.8).

Gender

	Male	Female
Cluster 1	34.3% (79)	65.7% (151)
Cluster 2	11.3% (26)	88.7% (204)

Cluster 3	13.0% (30)	87.0% (200)
Cluster 4	10.2% (37)	89.8% (324)
Cluster 5	42.6% (98)	57.4% (132)
Total	21.1% (270)	78.9% (1011)

Difference between groups χ^2_{4df}=136.0; P-value<0.0001.

Highest contribution to χ^2 value from Cluster 5 (66.1).

Residence

	Rural	Urban
Cluster 1	53.0% (122)	47.0% (108)
Cluster 2	99.1% (228)	0.9% (2)
Cluster 3	54.8% (126)	45.2% (104)
Cluster 4	35.5% (128)	64.5% (233)
Cluster 5	90.4% (208)	9.6% (22)
Total	63.4% (812)	36.6% (469)

Difference between groups χ^2_{4df}=338.4; P-value<0.0001.

Highest contribution to χ^2 value from Cluster 2 (126.6) and Cluster 4 (121.3).

Education

	University	Secondary school	Primary school	Literate	Illiterate
Cluster 1	28.3% (65)	33.5% (77)	33.0% (69)	8.3% (19)	0.0% (0)
Cluster 2	0.0% (0)	0.0% (0)	99.6% (229)	0.0% (0)	0.4% (1)
Cluster 3	6.1% (14)	19.6% (45)	16.5% (38)	32.6% (75)	25.2% (58)
Cluster 4	8.0% (29)	27.1% (98)	26.0% (94)	14.7% (53)	24.1% (87)
Cluster 5	7.8% (18)	17.8% (41)	24.8% (57)	18.7% (43)	30.9% (71)
Total	9.8% (126)	20.4% (261)	38.0% (487)	14.8% (190)	16.9% (217)

Difference between groups χ^2_{16df}=682.5; P-value<0.0001.

Highest contribution to χ^2 value from Cluster 2 (369.7).

Occupation

	Professional	Teacher	Skilled	Unskilled	Unemployed	Student	Retired	Homemaker

Cluster 1	11.7% (27)	2.6 (6)	7.8% (18)	25.2% (58)	21.3% (49)	21.7% (50)	6.1% (14)	3.5% (18)
Cluster 2	0.4% (1)	0.9% (2)	0.4% (1)	70.0% (161)	14.8% (34)	8.7% (20)	3.0% (7)	1.7% (4)
Cluster 3	6.5% (15)	19.6% (45)	25.7% (59)	17.4% (40)	9.6% (22)	7.4% (17)	6.1% (14)	7.8% (18)
Cluster 4	1.1% (4)	6.9% (25)	29.1% (105)	56.5% (204)	6.1% (22)	0.0% (0)	0.0% (0)	0.3% (1)
Cluster 5	0.0% (0)	0.0% (0)	32.2% (74)	58.7% (135)	2.6% (6)	3.5% (8)	1.3% (3)	1.7% (4)
Total	3.7% (47)	6.1% (78)	20.1% (257)	46.7% (598)	10.4% (133)	7.4% (95)	3.0% (38)	2.7% (35)

Difference between groups χ^2_{28df}=574.2; P-value<0.0001.

No Cluster χ^2 contribution to overall χ^2 value was higher than one third of the overall χ^2 value.

Marital status

	Married	Single	Separated	Widowed	Divorced
Cluster 1	58.3% (134)	33.5% (77)	4.8% (11)	1.7% (4)	1.7% (4)
Cluster 2	98.7% (227)	1.3% (3)	0.0% (0)	0.0% (0)	0.0% (0)
Cluster 3	64.8% (149)	22.2% (51)	3.9% (9)	7.0% (16)	2.2% (5)
Cluster 4	90.0% (325)	8.0% (29)	0.0% (0)	1.4% (5)	0.6% (2)
Cluster 5	54.3% (125)	45.7% (105)	0.0% (0)	0.0% (0)	0.0% (0)
Total	74.9% (960)	20.7% (265)	1.6% (20)	2.0% (25)	0.9% (11)

Difference between groups χ^2_{16df}=296.9; P-value<0.0001.

No Cluster χ^2 contribution to overall χ^2 value was higher than one third of the overall χ^2 value.

Table S3. Lifestyle of patients in the five clusters, and statistical analysis of the differences between clusters. Differences between proportions were assessed with the χ^2 test, clusters that deviated from the overall frequency distributions contributing to more than one third the overall χ^2 value were reported. Differences between means were assessed with the Analysis of Variance, with the F-test for the overall difference between clusters, and Dunn-Bonferroni post-hoc test for the difference between couples of clusters.

Nut alone

	No	Yes
Cluster 1	79.6% (183)	20.4% (47)
Cluster 2	78.3% (180)	21.7% (50)
Cluster 3	89.1% (205)	10.9% (25)
Cluster 4	60.1% (217)	39.9% (144)
Cluster 5	94.3% (217)	5.7% (13)
Total	78.2% (1002)	21.8% (279)

Difference between groups χ^2_{4df}=119.1; P-value<0.0001.

Highest contribution to χ^2 value from Cluster 4 (69.5).

BQ without tobacco

	No	Yes
Cluster 1	83.5% (192)	16.5% (38)
Cluster 2	100.0% (230)	0.0% (0)
Cluster 3	92.6% (213)	7.4% (17)
Cluster 4	69.5% (251)	30.5% (110)
Cluster 5	93.0% (214)	7.0% (16)
Total	85.9% (1100)	14.1% (181)

Difference between groups χ^2_{4df}=136.7; P-value<0.0001.

Highest contribution to χ^2 value from Cluster 4 (79.5).

BQ with tobacco

	No	Yes

Cluster 1	77.4% (178)	22.6% (52)
Cluster 2	45.7% (105)	54.3% (125)
Cluster 3	87.0% (200)	13.0% (30)
Cluster 4	74.8% (270)	25.2% (91)
Cluster 5	81.3% (187)	18.7% (43)
Total	73.4% (940)	26.6% (341)

Difference between groups χ^2_{4df}=121.9; P-value<0.0001.

Highest contribution to χ^2 value from Cluster 2 (90.5).

Pan masala

	No	Yes
Cluster 1	71.7% (165)	28.3% (65)
Cluster 2	92.2% (212)	7.8% (18)
Cluster 3	71.3% (164)	28.7% (66)
Cluster 4	96.4% (348)	3.6% (13)
Cluster 5	67.8% (156)	32.2% (74)
Total	81.6% (1045)	18.4% (236)

Difference between groups χ^2_{4df}=129.9; P-value<0.0001.

Highest contribution to χ^2 value from Cluster 4 (52.8).

Gutka

	No	Yes
Cluster 1	68.7% (158)	31.3% (72)
Cluster 2	80.4% (185)	19.6% (45)
Cluster 3	82.2% (189)	17.8% (41)
Cluster 4	99.2% (358)	0.8% (3)
Cluster 5	36.5% (84)	63.5% (146)
Total	76.0% (974)	24.0% (307)

Difference between groups χ^2_{4df}=317.1; P-value<0.0001.

Highest contribution to χ^2 value from Cluster 5 (197.0) and Cluster 4 (106.0).

Khara

	No	Yes
Cluster 1	96.5% (222)	3.5% (8)
Cluster 2	100.0% (230)	0.0% (0)
Cluster 3	99.6% (229)	0.4% (1)
Cluster 4	100.0% (361)	0.0% (0)
Cluster 5	100.0% (230)	0.0% (0)
Total	99.3% (1272)	0.7% (9)

Difference between groups χ^2_{4df}=31.4; P-value<0.0001.

Highest contribution to χ^2 value from Cluster 1 (25.4).

Others

	No	Yes
Cluster 1	97.0% (223)	3.0% (7)
Cluster 2	99.6% (229)	0.4% (1)
Cluster 3	78.3% (180)	21.7% (50)
Cluster 4	100.0% (361)	0.0% (0)
Cluster 5	100.0% (230)	0.0% (0)
Total	95.5% (1223)	4.5% (58)

Difference between groups χ^2_{4df}=195.7; P-value<0.0001.

Highest contribution to χ^2 value from Cluster 3 (157.6).

Total products

	1	2	3
Cluster 1	77.4% (178)	19.6% (45)	3.0% (7)
Cluster 2	96.1% (221)	3.9% (9)	0.0% (0)
Cluster 3	100.0% (230)	0.0% (0)	0.0% (0)
Cluster 4	100.0% (361)	0.0% (0)	0.0% (0)
Cluster 5	74.8% (172)	23.5% (54)	1.7% (4)
Total	90.7% (1162)	8.4% (108)	0.9% (11)

Difference between groups χ^2_{8df}=190.2; P-value<0.0001.

Highest contribution to χ^2 value from Cluster 5 (70.2).

Smokeless tobacco (SLT)

	No	Yes
Cluster 1	66.1% (152)	33.9% (78)
Cluster 2	83.0% (191)	17.0% (39)
Cluster 3	77.0% (177)	23.0% (53)
Cluster 4	85.9% (310)	14.1% (51)
Cluster 5	30.9% (71)	69.1% (159)
Total	70.3% (901)	29.7% (380)

Difference between groups χ^2_{4df}=238.1; P-value<0.0001.

Highest contribution to χ^2 value from Cluster 5 (171.7).

Smokeless tobacco (SLT) with slaked lime

	No	Yes
Cluster 1	84.8% (195)	15.2% (35)
Cluster 2	82.6% (190)	17.4% (40)
Cluster 3	70.4% (162)	29.6% (68)
Cluster 4	95.8% (346)	4.2% (15)
Cluster 5	93.5% (215)	6.5% (15)
Total	86.5% (1108)	13.5% (173)

Difference between groups χ^2_{4df}=91.0; P-value<0.0001.

Highest contribution to χ^2 value from Cluster 3 (50.8).

Smoking (cigarettes, beedi)

	No	Yes
Cluster 1	70.0% (161)	30.0% (69)
Cluster 2	64.8% (149)	35.2% (81)
Cluster 3	77.8% (179)	22.2% (51)
Cluster 4	79.8% (288)	20.2% (73)
Cluster 5	90.0% (207)	10.0% (23)
Total	76.8% (984)	23.2% (297)

Difference between groups χ^2_{4df}=49.1; P-value<0.0001.

Highest contribution to χ^2 value from Cluster 5 (22.5) and Cluster 2 (18.7).

Alcoholic drinks

	No	Yes
Cluster 1	78.3% (180)	21.7% (50)
Cluster 2	70.9% (163)	29.1% (67)
Cluster 3	74.8% (172)	25.2% (58)
Cluster 4	39.9% (144)	60.1% (217)
Cluster 5	71.7% (165)	28.3% (65)
Total	64.3% (824)	35.7% (457)

Difference between groups χ^2_{4df}=134.2; P-value<0.0001.

Highest contribution to χ^2 value from Cluster 4 (93.9).

BQ frequency

	Weekly	Daily
Cluster 1	95.2% (219)	4.8% (11)
Cluster 2	3.9% (9)	96.1% (221)
Cluster 3	11.3% (26)	88.7% (204)
Cluster 4	58.4% (211)	41.6% (150)
Cluster 5	7.4% (17)	92.6% (213)
Total	37.6% (482)	62.4% (799)

Difference between groups χ^2_{4df}=660.6; P-value<0.0001.

Highest contribution to χ^2 value from Cluster 1 (325.0).

BQ amount

	<5	5-9	10-15	>15
Cluster 1	63.5% (146)	27.8% (64)	7.8% (18)	0.9% (2)
Cluster 2	7.0% (16)	33.0% (76)	28.3% (65)	31.7% (73)
Cluster 3	35.7% (82)	30.0% (68)	21.3% (49)	13.5% (31)
Cluster 4	57.3% (207)	9.4% (34)	5.5% (20)	27.7% (100)
Cluster 5	73.5% (169)	6.5% (15)	7.4% (17)	12.6% (29)
Total	48.4% (620)	20.1% (257)	13.2% (169)	18.3% (235)

Difference between groups χ^2_{16df}=377.0; P-value<0.0001.

Highest contribution to χ^2 value from Cluster 2 (163.0).

BQ use

	Alone	With family	With friends	Mixed
Cluster 1	27.4% (63)	23.0% (53)	39.6% (91)	10.0% (23)
Cluster 2	0.0% (0)	0.4% (1)	96.5% (222)	3.0% (7)
Cluster 3	0.0% (0)	43.9% (101)	56.1% (129)	0.0% (0)
Cluster 4	0.3% (1)	2.8% (10)	97.0% (350)	0.0% (0)
Cluster 5	0.0% (0)	3.9% (9)	88.7% (204)	7.4% (17)
Total	5.0% (64)	13.6% (174)	77.8% (996)	3.7% (47)

Difference between groups χ^2_{12df}=672.4; P-value<0.0001.

Highest contribution to χ^2 value from Cluster 1 (314.3).

Swallow BQ juice

	No	Yes
Cluster 1	83.5% (192)	16.5% (38)
Cluster 2	37.8% (87)	62.2% (143)
Cluster 3	41.7% (96	58.3% (134)
Cluster 4	73.7% (266)	26.3% (95)
Cluster 5	9.6% (22)	90.4% (208)
Total	51.8% (663)	48.2% (618)

Difference between groups χ^2_{4df}=353.3; P-value<0.0001.

Highest contribution to χ^2 value from Cluster 5 (164.0).

Starting age (years)

	Mean (standard deviation)
Cluster 1	21.0 (5.7)
Cluster 2	29.1 (6.5)
Cluster 3	24.1 (5.1)
Cluster 4	22.4 (6.7)
Cluster 5	20.7 (7.9)

Total	23.3 (7.1)

Analysis of Variance. $F\text{-value}_{4df}=64.7$; $P\text{-value}<0.0001$

Dunn-Bonferroni post-hoc test

		Mean difference	P-value
Cluster 1	Cluster 2	-8.1	<0.0001*
Cluster 1	Cluster 3	-3.1	<0.0001*
Cluster 1	Cluster 4	-1.4	0.01
Cluster 1	Cluster 5	0.3	0.61
Cluster 2	Cluster 3	5.0	<0.0001*
Cluster 2	Cluster 4	6.7	<0.0001*
Cluster 2	Cluster 5	8.4	<0.0001*
Cluster 3	Cluster 4	1.7	0.002*
Cluster 3	Cluster 5	3.4	<0.0001*
Cluster 4	Cluster 5	1.7	0.002*

*statistically significant (P-value<0.005)

Use duration (years)

	Mean (standard deviation)
Cluster 1	9.7 (6.6)
Cluster 2	10.1 (6.1)
Cluster 3	6.7 (3.3)
Cluster 4	7.3 (4.9)
Cluster 5	8.6 (7.4)
Total	8.4 (5.9)

Analysis of Variance. $F\text{-value}_{4df}=16.8$; $P\text{-value}<0.0001$

Dunn-Bonferroni post-hoc test

		Mean difference	P-value
Cluster 1	Cluster 2	-0.4	0.47
Cluster 1	Cluster 3	3.0	<0.0001*
Cluster 1	Cluster 4	2.5	<0.0001*
Cluster 1	Cluster 5	1.1	0.03
Cluster 2	Cluster 3	3.4	<0.0001*
Cluster 2	Cluster 4	2.8	<0.0001*

Cluster 2	Cluster 5	1.5	0.004*
Cluster 3	Cluster 4	-0.6	0.22
Cluster 3	Cluster 5	-1.9	0.0004*
Cluster 4	Cluster 5	-1.3	0.006

*statistically significant (P-value<0.005)

Table S4. BQ Use Disorders (BUD) prevalence in the five clusters, and statistical analysis of the differences between clusters. Differences between proportions were assessed with the χ^2 test, clusters that deviated from the overall frequency distributions contributing to more than one third the overall χ^2 value were reported. Differences between means were assessed with the Analysis of Variance, with the F-test for the overall difference between clusters, and Dunn-Bonferroni post-hoc test for the difference between couples of clusters.

BUD categories

	No	Mild	Moderate	Severe
Cluster 1	10.0% (23)	17.0% (39)	29.6% (68)	43.5% (100)
Cluster 2	6.5% (15)	61.7% (142)	24.8% (57)	7.0% (16)
Cluster 3	0.4% (1)	6.1% (14)	21.3% (49)	72.2% (166)
Cluster 4	8.9% (32)	19.1% (69)	19.4% (70)	52.6% (190)
Cluster 5	3.9% (9)	9.1% (21)	19.1% (44)	67.8% (156)
Total	6.2% (80)	22.2% (285)	22.5% (288)	49.0% (628)

Difference between groups χ^2_{12df}=369.8; P-value<0.0001.

Highest contribution to χ^2 value from Cluster 2 (244.9).

BUD average score

	Mean (standard deviation)
Cluster 1	5.1 (2.7)
Cluster 2	3.1 (1.4)
Cluster 3	6.2 (1.7)
Cluster 4	5.4 (2.8)
Cluster 5	6.0 (2.1)
Total	5.2 (2.5)

Analysis of Variance. F-value$_{4df}$=68.4; P-value<0.0001

Dunn-Bonferroni post-hoc test

		Mean difference	P-value
Cluster 1	Cluster 2	2.0	<0.0001*
Cluster 1	Cluster 3	-1.1	<0.0001*

Cluster 1	Cluster 4	-0.3	0.16
Cluster 1	Cluster 5	-0.9	<0.0001*
Cluster 2	Cluster 3	-3.1	<0.0001*
Cluster 2	Cluster 4	-2.3	<0.0001*
Cluster 2	Cluster 5	-2.9	<0.0001*
Cluster 3	Cluster 4	0.8	<0.0001*
Cluster 3	Cluster 5	0.2	0.28
Cluster 4	Cluster 5	-0.6	0.001*

*statistically significant (P-value<0.005)

Reinforcement construct

	Mean (standard deviation)
Cluster 1	2.5 (1.2)
Cluster 2	2.3 (0.7)
Cluster 3	2.4 (1.0)
Cluster 4	2.4 (1.2)
Cluster 5	3.2 (1.2)
Total	2.5 (1.1)

Analysis of Variance. F-value$_{4df}$=25.5; P-value<0.0001

Dunn-Bonferroni post-hoc test

		Mean difference	P-value
Cluster 1	Cluster 2	0.2	0.05
Cluster 1	Cluster 3	0.1	0.43
Cluster 1	Cluster 4	0.03	0.78
Cluster 1	Cluster 5	-0.7	<0.0001*
Cluster 2	Cluster 3	-0.1	0.26
Cluster 2	Cluster 4	-0.2	0.07
Cluster 2	Cluster 5	-9.9	<0.0001*
Cluster 3	Cluster 4	-0.05	0.56
Cluster 3	Cluster 5	-0.8	<0.0001*
Cluster 4	Cluster 5	-0.7	<0.0001*

*statistically significant (P-value<0.005)

Social impairment

	Mean (standard deviation)
Cluster 1	1.2 (1.1)
Cluster 2	0.6 (0.7)
Cluster 3	1.7 (0.9)
Cluster 4	1.2 (1.1)
Cluster 5	0.8 (0.9)
Total	1.1 (1.0)

Analysis of Variance. F-value$_{4df}$=43.8; P-value<0.0001

Dunn-Bonferroni post-hoc test

		Mean difference	P-value
Cluster 1	Cluster 2	0.7	<0.0001*
Cluster 1	Cluster 3	-0.5	<0.0001*
Cluster 1	Cluster 4	0.02	0.77
Cluster 1	Cluster 5	0.4	<0.0001*
Cluster 2	Cluster 3	-1.1	<0.0001*
Cluster 2	Cluster 4	-0.6	<0.0001*
Cluster 2	Cluster 5	-0.3	0.006
Cluster 3	Cluster 4	0.5	<0.0001*
Cluster 3	Cluster 5	0.9	<0.0001*
Cluster 4	Cluster 5	0.4	<0.0001*

*statistically significant (P-value<0.005)

Stimulation construct

	Mean (standard deviation)
Cluster 1	0.8 (0.7)
Cluster 2	0.1 (0.4)
Cluster 3	1.0 (0.7)
Cluster 4	0.8 (0.7)
Cluster 5	1.3 (0.7)
Total	0.8 (0.8)

Analysis of Variance. F-value$_{4df}$=97.7; P-value<0.0001

Dunn-Bonferroni post-hoc test

		Mean difference	P-value

Cluster 1	Cluster 2	0.7	<0.0001*
Cluster 1	Cluster 3	-0.2	0.0001*
Cluster 1	Cluster 4	-0.05	0.33
Cluster 1	Cluster 5	-0.5	<0.0001*
Cluster 2	Cluster 3	-0.9	<0.0001*
Cluster 2	Cluster 4	-0.7	<0.0001*
Cluster 2	Cluster 5	-1.2	<0.0001*
Cluster 3	Cluster 4	0.2	0.0009*
Cluster 3	Cluster 5	-0.2	0.0001*
Cluster 4	Cluster 5	-0.4	<0.0001*

*statistically significant (P-value<0.005)

Pharmacologic symptoms

	Mean (standard deviation)
Cluster 1	0.7 (0.8)
Cluster 2	0.2 (0.5)
Cluster 3	1.1 (0.7)
Cluster 4	0.9 (0.5)
Cluster 5	0.7 (0.6)
Total	0.8 (0.7)

Analysis of Variance. F-value$_{4df}$=79.1; P-value<0.0001

Dunn-Bonferroni post-hoc test

		Mean difference	P-value
Cluster 1	Cluster 2	0.5	<0.0001*
Cluster 1	Cluster 3	-0.5	<0.0001*
Cluster 1	Cluster 4	-0.3	<0.0001*
Cluster 1	Cluster 5	-0.1	0.17
Cluster 2	Cluster 3	-0.9	<0.0001*
Cluster 2	Cluster 4	-0.7	<0.0001*
Cluster 2	Cluster 5	-0.6	<0.0001*
Cluster 3	Cluster 4	0.2	<0.0001*
Cluster 3	Cluster 5	0.4	<0.0001*
Cluster 4	Cluster 5	0.2	0.0004*

*statistically significant (P-value<0.005)

Table S5. Standard reference populations of 1,000 adult Indian BQ users according to gender, residence, and education, made using a large cross-sectional study investigating BQ prevalence among adult Indian individuals attending healthcare services.

Variable - category	BQ use Prevalence Ratio	Proportion (%)	Standard population size
Gender	1.92		
Males		65.8%	658
Females		34.2%	342
Residence	1.92		
Rural		65.8%	658
Urban		34.2%	342
Education	2.26		
Primary school diploma or lower		69.3%	693
Secondary school diploma or higher		30.7%	307

Table S6. Proportions of males and females, rural and urban residents, patients with lower and higher education with 95% confidence intervals (95CIs) and departure from the reference proportions of the standard populations of adult Indian BQ users (departure was present if the reference proportion did not fall within the 95CI with further 5% tolerance).

Variable	Proportion (%)	95CI	Reference (%)	Departure
Gender				YES
Males	21.1%	18.9-23.4%	65.8%	
Females	78.9%	76.6-81.1%	34.2%	
Residence				NO
Rural	63.4%	60.7-66.0%	65.8%	
Urban	36.6%	34.0-39.3%	34.2%	
Education				NO
Primary school diploma or lower	69.8%	67.2-72.2%	69.3%	
Secondary school diploma or higher	30.2%	27.8-32.8%	30.7%	

Table S7. Skewness and kurtosis of the categorical variables with more than two categories treated as ordinal (Normality assumed for skewness values within ±2 and kurtosis values within ±7).

	Number of categories	skewness	kurtosis	Normality
Age	5	0.11	-0.99	Yes
Education	5	0.09	-0.74	Yes
Occupation	8	0.57	1.17	Yes
Occupation	3	0.03	-0.02	Yes
Marital status	5	2.78	9.16	No
BQ use	4	-1.52	2.95	Yes
Total products	3	3.33	11.19	No
Starting age	continuous	0.98	1.72	Yes
Use duration	continuous	2.13	7.37	No
Amount	4	0.67	-1.09	Yes
BUD questionnaire score	continuous	0.09	-0.65	Yes

- The number of categories of the variable “Occupation” were originally eight, but three of them included <50 subjects; therefore, categories were then grouped into five (i.e., non-manual worker, manual worker, not working).
- The assumption of Normality of the variable “Marital status” was not met and the variable was transformed into binary (i.e., married, alone).
- One category of the variable “BQ use” included 77.8% of all subjects, while another category included <50 subjects; therefore, this variable was transformed into binary (i.e., without friends, with friends).
- The assumption of Normality for of variable “Total products” was not met and this variable was not considered for the multivariate analyses, as 90.7% subjects used only one product.
- The assumption of Normality of the variable “Use duration”, with a range of values 1-45 years, was not met, as 70.9% subjects had values ranging between 1 and 10 years, therefore, the variable was transformed into binary (i.e., 1-10 years, >10 years).

Table S8. Association of the explanatory variables with severe BUD. Unadjusted and adjusted odds ratios (OR's) with 95% confidence intervals (95CI's).

Variable	reference	unadjusted OR	95CI	adjusted OR	95CI
Age	18-25 years	0.78*	0.70-0.86	1.00	0.87-1.15
Gender	Female	0.59*	0.45-0.78	1.03	0.70-1.51
Residence	Rural	1.12	0.95-1.50	1.03	0.77-1.36
Education	University degree	1.13*	1.03-1.24	1.14*	1.02-1.27
Occupation	Non-manual working	0.72*	0.59-0.88	0.69*	0.55-0.86
Marital status	Married	2.09*	1.61-2.71	1.46*	1.05-2.05
BQ use	Without friends	0.55*	0.42-0.74	0.49*	0.35-0.67
Nut alone	No	0.82	0.63-1.07	1.41	0.85-2.34
BQ without tobacco	No	1.94*	1.40-2.68	2.74*	1.59-4.74
BQ with tobacco	No	0.34*	0.26-0.44	0.72	0.44-1.17
Pan masala	No	1.60*	1.20-2.12	1.80*	1.13-2.87
Gutka	No	1.79*	1.38-2.32	1.51	0.97-2.36
Khara	No	1.30	0.35-4.87	1.84	0.38-8.88
Other products	No	1.88*	1.09-3.26	2.32*	1.13-4.74
Starting age	10 years	0.94*	0.92-0.95	0.96*	0.94-0.98
Frequency	Weekly	0.84	0.67-1.06	0.85	0.64-1.12
Use duration	1-10 years	0.49*	0.37-0.64	0.48*	0.35-0.67
Amount	1-5 intakes	0.74*	0.67-0.81	0.79*	0.70-0.88
Swallow BQ juice	No	1.57*	1.26-1.96	1.78*	1.34-2.36
SLT	No	2.03*	1.59-2.59	1.97*	1.31-2.96
SLT with tobacco	No	0.95	0.69-1.31	1.51	0.96-2.36
Smoking	No	0.60*	0.46-0.78	1.08	0.74-1.58
Alcohol drinking	No	1.03	0.82-1.29	1.96*	1.37-2.82

The adjustment was made with multiple logistic regression analysis including all the explanatory variables.

*P-value<0.05

Table S9. Common Factor Analysis (CFA) of the explanatory variables and generated Factors. The characteristics of the generated Factors, called Profiles, were the explanatory variables with the highest Factor Loadings.

Correlations between explanatory variables (Pearson's r)

If more than one third of all r's showed P-value<0.05, the CFA was made to reduce the number of inter-correlated variables.

Total number of variables	23
Total number of r's	253
One third of all r's	83.3
Lowest number of required r's with P-value<0.05 for the application of CFA	84
Number of r's with P-value<0.05	145
Proportion of r's with P-value<0.05	57.3%

Eigenvalues of the generated Factors

Eigenvalue measures how much of the common variance of the original variables was explained by the generated Factors.

	eigenvalue	explained variance proportion
Factor 1	2.840	12.3%
Factor 2	2.063	9.0%
Factor 3	1.590	6.9%
Factor 4	1.375	6.0%
Factor 5	1.315	5.7%
Factor 6	1.271	5.5%
Factor 7	1.214	5.3%
Factor 8	1.187	5.2%
Factor 9	1.082	4.7%
Factor 10	1.039	4.5%

Factor Loadings (orthogonal rotation; Pearson's r)

Factor Loadings ≥0.4 (in red and bold characters) denoted high correlation with the Factor. Factor Loadings ranging between ≥0.15 and <0.4 (in red characters) denoted moderate correlation.

	Factor 1	Factor 2	Factor 3	Factor 4	Factor 5	Factor 6	Factor 7	Factor 8	Factor 9	Factor 10
Age	**0.784**	-0.084	0.118	-0.001	0.052	0.053	0.095	0.095	0.091	0.008
Gender	**0.794**	-0.028	0.030	-0.015	-0.043	0.075	0.019	-0.118	0.034	-0.009
Residence	-0.058	**-0.447**	0.157	-0.066	-0.154	-0.051	-0.008	**-0.443**	0.399	-0.028
Education	0.142	**0.584**	0.177	-0.245	-0.028	-0.056	-0.031	0.099	0.077	0.190
Occupation	-0.156	-0.045	0.103	0.033	-0.001	0.065	0.071	**0.829**	-0.009	0.040
Marital status	**-0.759**	-0.102	0.112	-0.046	0.064	0.009	-0.001	-0.0003	-0.038	-0.063

BQ use	0.034	0.305	**-0.462**	-0.095	0.034	-0.065	-0.131	0.045	0.124	0.364
Nut alone	0.109	-0.329	-0.192	**-0.614**	**0.455**	0.094	0.020	-0.061	0.071	0.350
BQ without tobacco	0.097	-0.127	-0.026	-0.091	**-0.881**	0.093	0.030	-0.022	-0.026	0.133
BQ with tobacco	0.112	-0.079	-0.077	**0.881**	0.215	0.041	-0.013	0.066	0.040	0.179
Pan masala	-0.067	0.025	-0.074	-0.102	0.103	-0.033	-0.092	0.045	-0.077	**-0.851**
Gutka	-0.310	**0.474**	-0.115	-0.145	-0.009	-0.337	0.080	0.039	-0.064	-0.122
Khara	-0.005	-0.094	-0.016	0.028	0.036	0.093	-0.057	-0.011	-0.008	0.114
Others	0.048	0.116	**0.848**	-0.049	0.022	0.012	-0.067	0.043	-0.011	0.128
Starting age	0.395	0.072	0.034	0.163	0.371	0.150	0.324	-0.048	-0.048	0.100
Frequency	0.031	**0.674**	0.029	0.188	0.222	0.066	0.033	-0.161	0.095	-0.097
Use duration	0.304	-0.057	-0.105	0.046	-0.067	0.002	-0.051	**0.505**	0.063	-0.322
Amount	0.135	0.104	-0.197	0.158	-0.113	**0.636**	-0.037	0.087	0.029	0.196
Swallow BQ juice	-0.131	**0.594**	-0.075	0.059	-0.067	0.239	-0.065	-0.038	-0.309	-0.037
SLT	-0.163	0.099	-0.010	0.036	-0.029	-0.355	**-0.451**	0.009	**-0.713**	-0.007
SLT with slaked lime	0.019	0.035	0.225	-0.138	0.049	**0.762**	-0.003	0.023	-0.048	-0.156
Smoking	0.083	-0.019	-0.032	-0.029	-0.012	-0.080	**0.921**	0.059	-0.055	0.057
Alcohol drinking	0.075	0.029	-0.102	0.051	0.031	-0.138	-0.237	-0.001	**0.851**	0.107

Final judgement. The CFA generated ten Factors corresponding to ten individual profiles with specific characteristics displayed in Table 6.

Table S10. Variance Inflation Factors (VIF's) between the explanatory variables. Multicollinearity was considered high for VIF-value>5, moderate for VIF-value>2.

Variable	R^2 value	VIF value
Age	0.421	1.7
Gender	0.388	1.6
Residence	0.254	1.3
Education	0.132	1.2
Occupation	0.098	1.1
Marital status	0.313	1.5
BQ use	0.108	1.1
Nut alone	0.660	2.9*
BQ without tobacco	0.586	2.4*
BQ with tobacco	0.676	3.1*
Pan masala	0.525	2.1*
Gutka	0.591	2.4*
Khara	0.035	1.0
Other products	0.319	1.5
Starting age	0.203	1.3
Frequency	0.230	1.3
Use duration	0.116	1.1
Amount	0.157	1.2
Swallow BQ juice	0.231	1.3
SLT	0.553	2.2*
SLT with tobacco	0.340	1.5
Smoking	0.383	1.6
Alcohol drinking	0.464	1.9

*moderate multicollinearity
